# Autophagy as a potential therapeutic target in regulating improper cellular proliferation

**DOI:** 10.3389/fphar.2025.1579183

**Published:** 2025-05-15

**Authors:** Prashant Kumar, Akash Choudhary, Sumit Kinger, Yuvraj Anandrao Jagtap, Vijay Kumar Prajapati, Deepak Chitkara, Subashchandrabose Chinnathambi, Rahul Kumar Verma, Amit Mishra

**Affiliations:** ^1^ Department of Biosciences and Bioengineering, Indian Institute of Technology Jodhpur, Jodhpur, Rajasthan, India; ^2^ Department of Biochemistry, University of Delhi South Campus, New Delhi, India; ^3^ Department of Pharmacy, Birla Institute of Technology and Science Pilani, Pilani, Rajasthan, India; ^4^ Department of Neurochemistry, National Institute of Mental Health and Neuro Sciences, Institute of National Importance, Bangalore, Karnataka, India; ^5^ Institute of Nano Science and Technology, Mohali, Punjab, India

**Keywords:** autophagy, autophagy inhibitors, autophagy activators, autophagy modulation cancer, cancer therapeutics

## Abstract

Autophagy is a degradative process that makes rapid turnover of old and impaired proteins and organelles possible. It is highly instigated by stress signals, like starvation, and contributes to the cell’s homeostasis. Autophagy performs a crucial function in keeping cell genomic integrity stable. Impaired autophagic flux is implicated in neurodegenerative diseases, abnormal ageing, and cancerous diseases. In diseases like cancer, autophagy performs a dualistic function; it can have both a tumor-suppressive and supportive role. Autophagy in the initial phases of tumorigenesis maintains the integrity of the genome and, if it fails, leads to cell death, thus having a tumor-suppressive role. Meanwhile, autophagy also imparts the function of the pro-survival mechanism in the latter stages of tumorigenesis and supports the cancerous cells in surviving conditions like hypoxia and increased oxidative stress. Autophagy also helps cancerous cells develop drug resistance in some cases. Thus, modulation of the autophagic mechanism is a possible therapeutic strategy in cancer therapy as its inhibition can sensitise cancer cells to anti-cancerous drugs. The promotion of autophagy, in some cases, can also safeguard cells from toxic protein aggregation and enhanced oxidative stress. Excessive autophagy can result in autophagic cell death. Autophagy also regulates several cellular processes and cell death pathways, like apoptosis. Therefore, an in-depth knowledge of the autophagy process and its regulating molecules is critically important. Pharmaceutical small molecules or cellular target modulation can help modulate the cellular autophagy process in the context of specific disease conditions.

## 1 Introduction

The term “autophagy” translates to “eating of oneself”, Christian de Duve in 1963 proposed the term, describing the presence of membraned (single or double) vesicles containing cytoplasmic contents and organelles in different stages of degradation (De Duve and Wattiaux, 1966). Some evidence suggests that the term autophagy was well used before 1963. A French physiologist named M. Anselmier used the term in the 1860s to refer to a phenomenon of a body’s self-nourishment system, which offers a person nourishment in extreme starvation by feeding on oneself (Ktistakis, 2017). Autophagy is a conserved and regulated process in cells of eukaryotes implicated in the lysosome-dependent recycling and degradation of cellular components impaired or senescent (Klionsky and Emr, 2000; Levine and Klionsky, 2004). Studies of autophagy in earlier days were based on morphological observations; therefore, the observations made were of later stages of autophagy before or after the lysosomal fusion with autophagosome. Using radioactive probes helped illustrate the earlier and intermediatory phases of autophagy (Gordon et al., 1988). Autophagy is a bulk degradative mechanism where constituents from cytoplasm are sequestered into an autophagosome (double-membraned) and transferred to lysosomal lumen for clearance (Yoshimori et al., 2004). As research on autophagy advanced, the selective forms of autophagy were also reported, where specific organelles (ER and mitochondria) or proteins are targeted for clearance (Bolender and Weibel, 1973; Lemasters et al., 1998; Beaulaton and Lockshin, 1977).

Besides cytoplasmic components, extracellular material and portions of the plasma membrane are also delivered to lysosomes through endocytosis (Tooze et al., 2014). The large and hydrophilic molecular cargoes need to be transported into the lumen of the lysosome *via* membrane barriers. Different forms of autophagy were observed that deliver these cargoes into the lysosomal lumen via membrane barriers. The macroautophagy, microautophagy, and chaperone-mediated autophagy (CMA) are major autophagy forms that have been well studied (Yang and Klionsky, 2010). In macroautophagy and microautophagy, the inner autophagosomal layer and intra-luminal-vesicle layer, respectively, cause the cargo to be exposed to lysosomal luminal hydrolysing enzymes, but in CMA, the substrate is directly translocated into the lumen through putative pores (Mizushima et al., 2008). The studies in the field of autophagy up to the 1990s were focused on biochemical, morphological, and physiological aspects of autophagy; the advancement in autophagy research then elucidated molecular elements of autophagy. Autophagy-related genes (ATGs) that are implicated in modulation and proper functioning of autophagy were identified (Tsukada and Ohsumi, 1993; Klionsky et al., 2003). More than 40 ATG genes are reported so far, of which 16 are core ATG genes. These genes are conserved and crucial for macroautophagy and other forms of autophagy (Furukawa et al., 2024; Levine and Kroemer, 2019).

The modulation of autophagy is controlled by various signalling pathways and molecular regulators (He and Klionsky, 2009). It is well established that this includes one such negative regulator of autophagy, especially in circumstances of nutrient abundance, referred to as mTOR pathway, having been shown to phosphorylate, thus inactivating the ULK1 complex. ULK1 is the core complex crucial for autophagosome formation. Either nutrient starvation or stress decreases the activity of mTOR, initiating autophagy (Saxton and Sabatini, 2017). The physiological pathway of activation of autophagy through AMPK signalling necessitates instigation of ULK1 complex and a reduced state of mTOR activity, which are generated by reduced cellular energy levels (Alers et al., 2012). This complex, class III phosphatidylinositol 3-kinase (PI3-K), comprises proteins Beclin-1, ATG14L, and VPS34; it participates in the production of autophagosomes that are further mediated by other proteins, including Bcl-2. Bcl-2 can interact with Beclin-1 and functions as a negative modulator for autophagy (McKnight and Yue, 2013; Kang et al., 2011; Funderburk et al., 2010). In addition, transcriptional mechanisms that involve transcription factors such as TFEB, FOXO, and p53 affect levels of many autophagy-associated genes in response to various stress stimuli (Di Malta et al., 2019).

Furthermore, under intracellular nutrient starvation conditions, autophagy is a significant source of intracellular amino acids, lipids, and other key molecules that result from breakdown of cellular constituents. This is a key process for upkeep of the cell’s energy supply and for the continuation of cellular function (He, 2022). It is absolutely crucial for clearing impaired organelles, misfolded proteins, and potentially harmful cellular debris (Gómez-Virgilio et al., 2022). Autophagy holds a substantial part in processes of embryogenesis and differentiation and in elimination of surplus cellular components to reorganize cellular structures (Tettamanti et al., 2019). Moreover, autophagy interacts with immune system by involving in the clearance of intracellular pathogens, antigen presentation to immune cells, and also in inflammatory responses (Deretic et al., 2013). Apart from its degradative functions, autophagy and its associated molecules are engaged in a whole array of other physiological activities (Wang and Qin, 2013). The proper physiological function of autophagy is necessary to upkeep cell’s homeostasis and to respond to stressors (Gómez-Virgilio et al., 2022). Many physiological and pathological disease states are related to autophagy, including dysfunctional ageing, neurodegenerative diseases, infections, and cancer (Ortega et al., 2024).

The impaired autophagy mechanism is reported in numerous disorders and pathological conditions (Khandia et al., 2019). There are reports present that suggest a positive role of autophagy in cellular proliferation (Wang et al., 2020). Improper or excessive cellular proliferation is a hallmark of cancer (Holland, 2010). There are reports present suggesting that autophagy downregulation in cancer may inhibit proliferation (Wei et al., 2011; Sheng et al., 2018). The mechanistic details of autophagy in regulating cellular proliferation are not fully elucidated, although there is some evidence present suggesting some cell cycle proteins can modulate autophagy and *vice versa* (Zheng et al., 2019). This complex autophagy role is also observed based on the different types and stages of tumors; it may exhibit tumor suppressor characteristics or promote survival of tumors (Bhutia et al., 2013). Therefore, though autophagy eliminates the damaged organelles and proteins that may promote tumorigenesis, tumor cells can use autophagy to sustain stressors like nutrient starvation (Mathew et al., 2007). Impaired autophagy flux is reported in various neuronal degenerative disorders like Alzheimer’s, Huntington’s, and Parkinson’s, where failure of autophagy causes inadequate clearance of misfolded polypeptides and impaired organelles in neurons and leads to neuronal dysfunction and degeneration (Guo et al., 2018). Metabolic diseases are also linked with autophagy impairment, such as obesity, diabetes, and liver diseases, as autophagy coordinates lipid metabolism, insulin sensitivity, and mitochondrial functions (Jakubek et al., 2024).

## 2 Overview of the autophagy process

**FIGURE 1:**
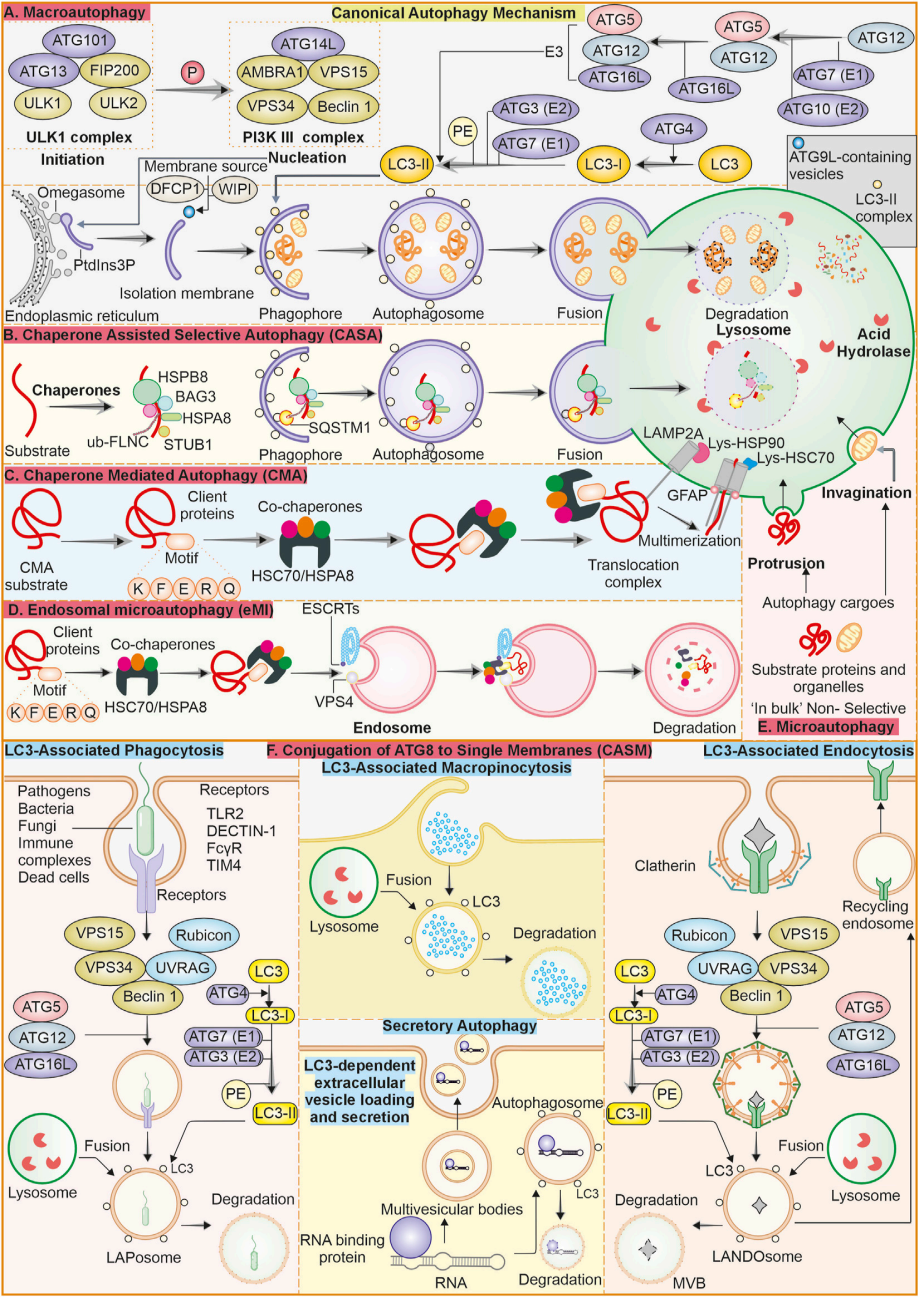
Overview of the autophagy process and its various types: **(A)** Macroautophagy: Macroautophagy is a well-described canonical form of the bulk degradation process. It initiates with forming of a phagophore, that closes and forms an autophagosome, which further fuses with the lysosome to clear its contents through degradation. **(B)** Chaperone-assisted selective autophagy (CASA): Selective autophagy where chaperones like HSPB8, BAG3 and HSPA8 identify the cargo and sequester into autophagosome and later fuse with lysosome for degradation. **(C)** Chaperone-mediated autophagy (CMA): This form of autophagy, with HSPA8 chaperone and co-chaperones selectively targets the substrate having a KFERQ motif, and this system transfers the substrate to the lysosome lumen for degradation through LAMP2A. **(D)** Endosomal microautophagy (eMI): It is similar to CMA and depends on chaperones and co-chaperones to selectively identify the cargo; after that, it enters the late endosome for degradation instead of the autophagosome. **(E)** Microautophagy: This form of autophagy does not require significant machinery. Here, the lysosomal membrane either invaginates or protrudes to engulf the cargo and transfer it to the lumen, where hydrolyzing enzymes are present. **(F)** Conjugation of ATG8 to single membranes (CASM): ATG8 family proteins, when conjugated to single membrane vesicles and internalize the cargo, is collectively called CASM. There are different forms of these non-canonical autophagy mechanisms like LC3-associated phagocytosis, LC3-associated macropinocytosis and LC3-associated endocytosis. There is one secretory form of autophagy called LC3-dependent extracellular vesicle loading and secretion, which is involved in the packaging and export of RNA and proteins.

As discussed above, autophagy is a very well-modulated mechanism strictly conserved from yeast to humans (Delorme-Axford and Klionsky, 2018). It has received much attention due to its roles in several physiological processes and its implications in many disorders, like cancer (Ryter et al., 2013). Well-characterised form of autophagy is macroautophagy, which utilises vesicles called autophagosomes (double-membrane) to sequester cytoplasmic contents like impaired organelles, misfolded proteins and other cellular debris (Levine and Kroemer, 2008). Macroautophagy is regulated mainly through some complexes and systems. The PI3K complex, the ULK1 complex, and the ATG8 system are among them. Specific sensor molecules like mTOR and AMPK modulate the processes of autophagy. The PI3K complex is essential in autophagy initiation as it is involved in forming the autophagosome. The ULK1 complex (serine/threonine kinase) is instigated upon stress like starvation and works downstream of the mTOR pathway, further facilitating onset of autophagy by phosphorylation of proteins related to autophagy. After the phagophore is formed, elongation and closing of autophagosome membrane are facilitated by the ATG8 system, which consists of LC3 and GABARAPs. Overall, these complexes and molecules form the core machinery, which is crucial for coordinating the autophagy process to ensure the preservation of homeostasis and proper stress response (Feng et al., 2014). After the formation of the autophagosome, it sequesters the cytoplasmic materials and matures; after that, autophagosome is fused with lysosome, initiating the clearance of contents in presence of hydrolysing enzymes. This is vital for degrading damaged organelles, proteins, and cellular health Figure 1 (Wang et al., 2003).

In contrast, microautophagy is defined by the protrusion or invagination of lysosomes membrane and engulfing of substarte inside lumen (Mijaljica et al., 2012; Wang et al., 2023). Lysosomes’ direct uptake of cellular contents represents a central factor ensuring cell homeostasis at basal conditions. Endosomal microautophagy (eMI) is a unique autophagy type characterized by late endosomes direct internalisation of cytoplasmic components by membrane’s invaginations, thus eliminating the use of autophagosomes (Olsvik et al., 2019). This mechanism is vital for cellular homeostasis because it selectively degrades damaged proteins, organelles, and other cytoplasmic components. It, therefore, regulates many cellular processes, such as immune response and cell signalling. eMI ensures efficient recycling of cellular components and maintains cellular health and functionality. eMI can be bulk or selective as it relies on interaction of hsc70 with KFERQ-like motif present in substrate to translocate them to the LE/MVB membrane by interaction of phosphatidylserine. The machinery for ESCRT consists of Vps4 and Tsg101, which aids in the internalization of substrates into ILV. The substrates may get degraded within the LE/MVB or after the LE/MVB fuses with the lysosome (Morozova et al., 2016; Sahu et al., 2011).

The selective type of autophagy is CMA, where specific proteins are targeted for degradation (Dice, 2007). This process includes the chaperone Hsc70’s identification of motif KFERQ-like sequence in targeted protein. Targeted protein is translocated across lysosome’s membrane by receptor lysosome-associated membrane protein 2A (LAMP-2A), where it is degraded (Majeski et al., 2004). There is another autophagy type called Chaperone-assisted selective autophagy (CASA), which is a process that focuses on selective degradation of damaged and misfolded proteins. The core CASA complex consists of three central proteins: HSPA8, HSPB8, and BAG3, all of which are crucial for maintaining proteostasis in neurons and muscle cells. Mutations in such genes lead to diseases ranging from (cardio) myopathies to neurodegenerative diseases (Tedesco et al., 2023).

## 3 Non-canonical autophagy

In macroautophagy, the double-membraned autophagosome formation and maturation depend on conjugation of ATG8 proteins like GABARAPs and LC3s to phosphatidylethanolamine (PE) (Ichimura et al., 2000; Johansen and Lamark, 2020). Various studies have also reported that ATG8 has autophagy-independent functions (Galluzzi and Green, 2019; Nieto-Torres et al., 2021). It is also noted that ATG8 can be recruited by phosphatidylserine (PS) into single membrane vesicles, termed Conjugation of ATG8 to single membranes (CASM) (Durgan et al., 2021). The studies have suggested that parallel functions of ATG8 proteins leading to either cargo degradation or secretion are called non-canonical autophagy (Nieto-Torres et al., 2021; Codogno et al., 2012). LC3-associated phagocytosis (LAP) combine phagocytosis with LC3 protein recruitment, thus facilitating the LAPosomes fusion with lysosomes to improve the clearance and removal of pathogens within LAPosomes. It differs from xenophagy, in which intracellular pathogens, as well as other foreign material, are targeted for degradation (Herb et al., 2020). Both LAP and xenophagy utilize the same LC3 conjugation machinery, including ATG3, ATG7, and an ATG12-ATG16L1-ATG5 complex. However, the mechanism of induction is different; generation of PI(3)P on membranes, involvement of ROS, and conjugation of LC3 to membranes (single) are all different features of LAP. Some components of the PI3K complex are shared between LAP and autophagy, but recruitment of LC3 requires Rubicon and UVRAG proteins (Heckmann and Green, 2019).

LC3-associated endocytosis, or LANDO, is initiated by the identification of substrate through TREM2 and TLR (cell surface receptors), consequently internalizing clathrin-mediated endosomes. Process of LC3 recruitment to LANDOsome is parallel to LAP. Unlike LAP, LANDO is characterized by multiple endpoints that include fusion of the LANDOsomes with the lysosome, clearing of the ligand, and the cell surface recptor is recycled back to membrane (Heckmann et al., 2019). LC3-dependent extracellular vesicle loading and secretion (LDELS) is a type of secretory autophagy. It is reported as LC3 conjugation machinery mediating incorporation of cargo into the secretory vesicles that are exported outside the cell. Reports have shown that RNA-binding proteins are major cargos for this process, affecting non-coding RNAs (ncRNAs) and snoRNA secretion. This pathway requires ATG proteins and Rab27a to prevent the accumulation of receptors for autophagic cargo (Leidal et al., 2020; Eng et al., 2021). Cells react to damage of the plasma membrane by instigating macropinocytosis-related proteins at the injury site. Following sealing, they produce large macropinosomes at the repair site, which are marked with LC3B protein. The process is reliant on Rubicon, ATG7, and p62. Shrinking of the internalized macropinosome by osmotic draining and fusion with lysosomes. This is reported as a form of macropinocytosis, LC3-associated macropinocytosis (LAM), which helps remove damaged materials and restore membrane integrity (Sønder et al., 2021).

## 4 Autophagy implicated in diseases associated with cellular proliferation

The process of cellular proliferation is crucial for cell growth and division, as well as tissue repair and development. Recently, there have been several reports suggesting roles of autophagy in tissue repair and regeneration (Moreno-Blas et al., 2025). Excessive or improper cellular proliferation may become a risk factor for several diseases, ranging from cancer to inflammatory diseases. Mutations in oncogenes and tumor suppressor genes create an imbalance between cell death and cell division, resulting in excessive cellular proliferation (Dakal et al., 2024). RAS and MYC are oncogenes, and certain muations in these genes result in promotion of prolonged proliferation in absence of any external stimuli. In RAS-driven pancreatic, colorectal and lung cancers, autophagy helps in maintaining tumor growth by metabolic reprogramming and maintaining mitochondrial integrity, as can be observed in autophagy-deficient RAS mutant cells accumulating impaired mitochondria and impaired energy production. ATG7 deletion in BRAF V600E-driven lung tumors, mitochondrial impairment, along with metabolic dysfunction is observed (Ariosa et al., 2021; Mathiassen et al., 2017). In murine models with ATG16L1 (keratinocyte-specific), deletion exacerbated the skin inflammation and tumorigenesis. The deficiency of autophagy in these keratinocytes sensitizes them towards TNF-dependent apoptosis, altering hair follicle stem cell activation and wound healing (Van Hove et al., 2023). In liver fibrosis, the activation of hepatic stellate cells (HSC) is necessary. Autophagy in HSC instigates their transdifferentiation into myofibroblasts (collagen secreting) by degrading lipid droplets. Autophagy inhibition reduced the deposition of collagen in mouse models (Chen et al., 2025; Lucantoni et al., 2021). In chronic kidney diseases role of autophagy is observed where in renal fibroblasts, autophagy drives extracellular matrix production through TGF-β/SMAD4 signalling (Ruby et al., 2023 Lucantoni et al., 2021). The autophagy deficiency also impairs the clearance of bacteria, which exacerbates inflammation, whereas TNF-α inhibition may restore autophagy, resulting in reduced keratinocyte proliferation. CD4^+^ T cells in rheumatoid arthritis show increased autophagy, which supports their survival and inflammatory activities. In collagen-induced models, ATG5 knockout reduced the T cell proliferation and alleviated arthritis (Lin et al., 2024; Zhao et al., 2024).

Autophagy is implicated in various other cellular processes, and thus, its role in other disease models also varies. Some of the disease models that are extensively researched are cardiovascular diseases, neurodegenerative disorders, metabolic diseases, infectious diseases, cancer and congenital disorders. In models of cardiovascular diseases, *ATG5/ATG7* knockout mice pressure overload (TAC) was utilised, showing dual role of autophagy where at basal level, autophagy maintains homeostasis and dysregulation causes exacerbated heart failure (Schiattarella et al., 2016; Nishida et al., 2009). Autophagy is involved with maintaining the cardiomyocyte health during the stress conditions but becomes detrimental when it is dysregulated. During myocardial ischemia, autophagy is reported to clear the damaged organelles and mitigate ROS production and apoptosis (Jiang et al., 2023). Pressure overload model studies showed that autophagy clears the polyubiquitinated proteins in cardiomyocytes, which results in prevention of accumulation of toxic aggregates. Mutant α-B-crystallin in desmin-associated cardiomyopathy disrupts the proteostasis, autophagic clearance of aggregates delays heart failure (Hill, 2011). Models of neuronal degenerative disorders studies in SENDA patients and a Pink1/Prkn Knockout mouse model of Parkinson’s autophagy were observed to prevent protein aggregation, and the impairment of autophagy caused neuronal death (Kingwell, 2013; Klionsky et al., 2021). Autophagy is also indicated to be disturbed in metabolic disorders like type 2 diabetes, as it has a protective role on β-cell functioning and insulin sensitivity during stress conditions. Studies on ATG7 haploinsufficient mice model reported an exacerbated intolerance towards glucose and hepatic steatosis under high-fat diet. This observation associates deficiency of autophagy to the progression of diabetes (Park et al., 2022). In congenital disorders, mutation in autophagy-related genes may cause multi-system disorders. For instance, in Vici syndrome, the EPG5 mutation disrupts autophagosome and lysosome fusion, resulting in cardiomyopathy and neurological disorders (Dafsari et al., 2025). WDR45 defects may impair the autophagy in neuronal cells causing SENDA to have Parkinsonian features (Kingwell, 2013). In infectious diseases, like HIV infection, autophagy is reported to clear the viral components; furthermore, it is also subverted by virus-promoting persistence. The HIV-1 Tat protein reduces the maturation of autophagosome while the Nef also impairs autophagy, which further enables viral replication (Lamsira et al., 2025). A list of autophagy related molecules and their roles in cellular proliferation is presented in Table 1.

**TABLE 1:** Role of autophagy related molecules in cellular proliferation.

Molecule	Role in autophagy	Mechanism in cell proliferation	Cell lines/tissues	References
Beclin 1	Forms PI3K complex, tumor suppressor	Decrease proliferation	Human MCF7 breast carcinoma cells Cervical cancer HeLa cells	Liang et al. (1999), Wang et al. (2007)
mTOR	Autophagy inhibition through Phosphorylating ULK1 and ATG13	Translation of proteins that regulate proliferation	Mouse embryonic fibroblasts	Dowling et al. (2010)
AMPK	Activates autophagy during energy stress by phosphorylating ULK1	Suppresses cellular proliferation	LN229 glioblastoma cells	Zhang et al. (2019), Motoshima et al. (2006)
ULK1/2	Autyophagy initiation by phosphorylating downstream targets	Suppresses cellular proliferation	HEK293T and HeLa cells and MEFs	Jung et al. (2011)
LC3	Processed LC3-II embeds in autophagosome membrane	High LC3B is associated with proliferation	Tissue specimens from breast carcinoma, melanoma and, other cancers	Lazova et al. (2012)
ATG5	Autophagosome elongation	Knockout of Atg5 inhibits proliferation	DF-1 cells	Liao et al. (2019)
ATG12	Autophagosome elongation	Overexpression of ATG12 suppresses proliferation	HCC tissues	Wang et al. (2024)
p62	Autophagy receptor linking ubiquitinated cargo with LC3	Promotes proliferation	Prostate tumor tissues	Jiang et al. (2020b)
Vps34	Phagophore membrane nucleation	Inhibition of Vps34 impairs migration	Breast cancer cell lines MCF-7 and MDA-MB-231	Di Donato et al. (2022)
Bcl-2	Interacts with Beclin-1 and inhibit autophagy under normal conditions	Decreases cell proliferation	SKOV3 cell line (human ovarian carcinoma)	Belanger et al. (2005)
ATG16L1	Required for LC3 lipidation	Atg16L1 deficiency increased T cell proliferation	Atg16L1^HM^ and Atg16L1^flox/flox^ mice	Hubbard-Lucey et al. (2014)
TSC2	GTPase activating protein for Rheb	Loss of TSC2 increased proliferation	TSC2-null ELT3 cell and MEFs	Goncharova et al. (2011)
Rheb	Small GTPase	Silencing of RHEB inhibits cell proliferation	CRC cell lines including HT29, HRT18, SW620, and HCT116	Tian et al. (2020)
AMBRA1	Links ULK1 and Vps34 complex	Loss of a single Ambra1 allele is sufficient to increase cell proliferation	HEK293, MEFs and HeLa cells	Cianfanelli et al. (2015)
HS1BP3	Negative regulator of autophagosome biogenesis	Silencing of HS1BP3 inhibits proliferation	HCC cell lines (HUH-7, SUN-449, HLE, and SMMC-7721)	Hu et al. (2022)
p53	Nuclear p53 instigate autophagy and cytoplasmic p53 inhibits autophagy	Mutant p53 protein is associated with high tumor proliferation	Breast cancers from axillary lymph node-negative patients	Allred et al. (1993)
PTEN	Inhibits PI3K/Akt/mTOR pathway	Inhibit cell proliferation	A549 Cells	Lu et al. (2016)
HMGB1	Promotes autophagy by displacing Bcl-2	Induces proliferation	Bovine aorta endothelial cells Mesoangioblast cell lines	Palumbo et al. (2004)
RAC3/SRC3	Transcriptional co-activators regulating autophagy gene	Induces proliferation	Prostate cancer tissue	Zhou et al. (2005)
LAPTM4B	Lysosomal protein promoting autophagy	Induces proliferation	T24 and 5637 bladder tumor cells	Yin et al. (2021)
BRAF	Modulates autophagy via MEK/ERK signaling	BRAF V600E mutant maintains proliferation	Human PTC-derived cell lines KAT5 and KAT10	Liu et al. (2007)
c-MYC	Transcription factor inducing autophagy genes	c-MYC expression promoted high proliferation	Mesenchymal stromal cells	Melnik et al. (2019)

## 5 Autophagy functioning in tumor suppression

**FIGURE 2:**
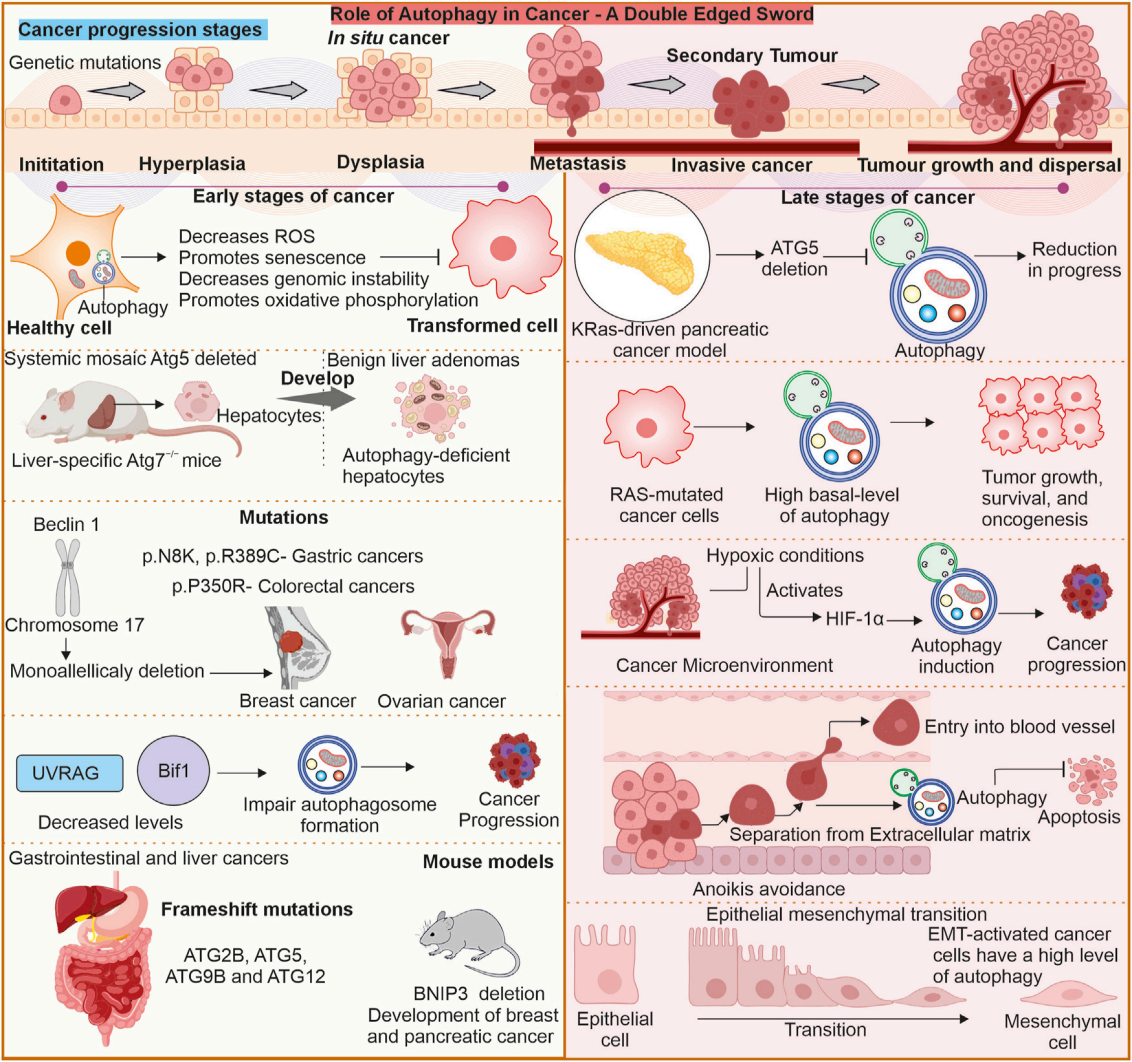
Role of autophagy in tumor proliferation a double-edged sword: In cancer autophagy can play dualistic role. It can have anti-proliferative role in early stages of cancer progression. The deletion or mutation of various ATG genes can support spontaneous tumor generation suggesting their anti-proliferative role. Similarly, once tumor is established autophagy supports the tumor cells by providing protection against stress conditions like hypoxia.

Autophagy plays several roles in the mechanisms involved in both suppression of cancer initiation and progression. Among its more significant functions in the context of preventing cancer, the ability to upkeep cell’s homeostasis in stressful conditions is essential (Vitto et al., 2022). The degradation and recycling of impaired organelles and polypeptides help in avoiding build-up of damages that precede instability of genome as a potential causative agent in cancer (Miller and Thorburn, 2021; Vessoni et al., 2013). Autophagy promotes cell survival in adverse conditions, including nutrient starvation, hypoxia, and oxidative stress, which contribute to uncontrolled tumorigenicity (Mizushima et al., 2008; Rakesh et al., 2022; Lee et al., 2012). Autophagy clears potentially oncogenic material and maintains cellular homeostasis; autophagy in the initial stages of tumorigenesis guards against cancer initiation (Patergnani et al., 2021). Autophagy is also said to regulate cell death pathways by ensuring that apoptosis is controlled and prevents the uncontrolled proliferation of cells, a characteristic of cancer (Verma et al., 2021; Liu et al., 2023). Enhanced excessive autophagy can cause autophagic cell death when the cells get extensively damaged or under stress. Cell death *via* autophagy removes damaged or potentially malignant cells that should not contribute to accumulating additional mutations leading to cancer cells (Kanzawa et al., 2005). Functional autophagy in cellular quality control is such that only healthy cells are maintained to restrain the possibility of carcinogenesis Figure 2 (Yang et al., 2011; Linder and Kögel, 2019).

The inhibition or downregulation of autophagy may promote tumorigenesis. Some observations have been reported where this association between autophagy and tumorigenesis is established (Takahashi et al., 2007). The ATG knockout models show role of ATGs and Beclin-1, as well as autophagy inhibition, in promoting a higher incidence of tumorigenesis. Tumor cells, under stressful conditions, can escape primary tumor through metastasis and invasion. In primary tumor autophagy is associated with tumor cell necrosis and inflammation inhibition (Kenific et al., 2010). Autophagy is also involved in suppression of epithelial-to-mesenchymal transition (EMT) by degrading p62 and TWIST1, an EMT transcription factor (Qiang et al., 2023). Autophagy has a dualistic role in cancer, and thus it can either enhance or reduce the invasion and metastasis of tumor cells (Lisanti et al., 2010). Initially, autophagy enhances cancer cell survival by reducing necrosis and also reduces the infiltration of macrophages (Mowers et al., 2018; DeNardo et al., 2009; Bingle et al., 2002; Xia et al., 2020). Autophagy can also enhance cancer cell invasion and migration (Sharifi et al., 2016). Chaperone-mediated autophagy is also associated with tumor cell metastasis, as CMA inhibition results in reduced metastasis *via* the downregulation of migration and anoikis resistance (Kon et al., 2011). In breast cancer, there is an association between CMA and metastasis (Han et al., 2017). CMA is reported to clear HSD17B4 protein implicated in cellular invasion and migration (Zhang et al., 2017). Downregulated CMA by LAMP2A modulation may reduce or inhibit the tumor growth (Han et al., 2017).

There are evidence present that suggest that in many cases of cancer, the autophagy process is compromised. The earlier evidence came from studies on hemizygous mouse models for Beclin-1, which are prone to tumor development. Human BECN1 gene encodes for Beclin-1 protein, which is needed for autophagy, is located on chromosome 17q21, with monoallelic deletions reported in breast malignancies and ovarian and prostate cancers (Aita et al., 1999). The experiment on breast cancer cells also elucidated a frequent loss in Beclin-1 (Aita et al., 1999; Liang et al., 1999; Yue et al., 2003). Apart from this, the deletion of ATG7 can trigger tumorigenesis in the liver (Takamura et al., 2011). Autophagy loss in the liver may promote the generation of hepatocyte-derived progenitor cells involved in tumor initiation (Barthet et al., 2021). This evidence suggests that impairment of autophagy may help establish the tumor cells. Nevertheless, it is still unknown whether the suppression of autophagy is responsible for tumorigenesis or provides the microenvironment for tumorigenesis. Tumor suppressor transcription factors like p53 also interact with autophagy pathways. The autophagy modulation by p53 can be either negative or positive. p53, which induces cell cycle arrest and apoptosis after detecting damage to DNA, can increase autophagy (Rahman et al., 2022). At the basal level, p53, when in cytoplasm, suppresses autophagy (Tasdemir et al., 2008).

The point mutations in Beclin-1 are rare, and some mutations like p. N8K, p. R389C in gastric and p. P350R colorectal cancer are observed (Lee et al., 2007). Bif-1, or Endophilin B1, interlates with Beclin 1 through UVRAG as well as is a positive modulator of PI3KC3. In conditions like starvation, Bif-1 localises to autophagosomes in response, colocalising with ATG5 and LC3. Moreover, the absence of Bif-1 inhibits autophagosome production. It is reported that the knockout models of Bif-1 can increase the probability of spontaneous tumor generation (Takahashi et al., 2007). Similarly, UVRAG also imparts an essential function in suppressing tumorigenesis. A frameshift mutation in UVRAG is reported in cancer conditions expressing UVRAG^FS^. The Mice model with UVRAG^FS^ was reported to have spontaneous tumorigenesis (Quach et al., 2019). Autophagy-related gene 4 (ATG4) protein, a crucial protein for autophagy process which, is directly implicated in the forming of autophagosomes. The ATG4 protein imparts a very important role in processing and lipidating LC3, which is necessary for the proper maturation of autophagosomes (Satoo et al., 2009). Recently, an important connection between ATG4B and the progression of cancer has been observed. More and more evidence indicates the overexpression of ATG4B in various cancer types, which makes it a prospective candidate for anticancer therapeutic strategies (Fu et al., 2019). Some reports also suggest that frameshift mutations in ATG genes cause cancer of colorectal and gastrointestinal (Kang et al., 2009). Other studies suggest that deletion of BNIP3 can cause breast cancer (Chourasia et al., 2015).

## 6 Role of autophagy in tumor promotion

The autophagy process is also reported to promote the established tumor’s progression and survival. The reported increased LC3 puncta levels and autophagosome accumulation in some tumor tissue suggest the increased autophagy supporting tumorigenesis (Fujii et al., 2008). Increased autophagy in cancer cells may support adaptation to harsh microenvironment of an established tumor characterised by nutrient scarcity, hypoxia, and oxidative stress (Yang et al., 2015). Autophagy delivers intracellular building blocks from degrading and recycling the components inside the cell, forming a principal source requisite for cell survival and proliferation. Thus, it helps sustain cancer cells’ metabolic needs in stress conditions (Karantza-Wadsworth et al., 2007; Degenhardt et al., 2006). In the hypoxic regions of tumors, autophagy helps remove damaged mitochondria *via* mitophagy, decreasing ROS quantity and preventing cell death (Zaarour et al., 2021). Consequently, this survival mechanism allows cancerous cells to sustain in microenvironment and promote tumor development (Poillet-Perez et al., 2015).

The autophagy involvement in cancer cell proliferation has been highlighted by multiple studies. In RAS-mutant cancer cases, autophagy is essential for providing glutamine by degrading various cellular components. This glutamine is converted to α-ketoglutarate through glutaminase (Vitto et al., 2022; Chavez-Dominguez et al., 2020). Mitophagy prevents the accumulation of ROS, thus maintaining the mitochondrial health required for proliferation in pancreatic ductal adenocarcinoma (Vitto et al., 2022; Mowers et al., 2018). Autophagy is also involved in anoikis resistance. During the metastasis phase, detached cancer cells evade anoikis, which is detachment-induced apoptosis caused by the degradation of pro-apoptotic signals by autophagy (Tonkin-Reeves et al., 2023; Sui et al., 2013). Apart from supporting cancer cell survival and metastatic spread, autophagy also increases their motility. Autophagy clears proteins like paxillin through LC3 interaction. Paxillin is a focal adhesion protein, enabling disassembly of cell matrix adhesion which further facilitates migration in breast cancer and melanoma (Mowers et al., 2018). Similarly, in TGF-β-induced autophagy instigates EMT in non-small cell lung carcinoma, further enhancing invasive potential (Hwang et al., 2022). Autophagy also helps in evading immune surveillance as it reduces MHC-I expression on tumor cells, which results in impaired CD8^+^ T cells recognition (Bustos et al., 2020).

In addition, autophagy is a significant aspect that enhances the survival of cancer stem cells (CSCs). CSCs have capabilities of differentiation and self-renewal, forming a subpopulation of cells. These cells are associated with recurrence, metastasis of tumors, and resistance of cancers to primary therapies. Autophagy supports CSC metabolic modulation by providing them with all the required nutrients and energy for survival and proliferation. Autophagy through the maintenance of stemness and survival of CSC contributes to tumor heterogeneity and the aggressive nature of the cancers (Rahman et al., 2024; Wang et al., 2022). Studies on mice have given proof of complex functioning of autophagy in cancer development. Reports show that mice with an ATG5 gene mosaic deletion develop non-cancerous liver adenomas, which do not form malignant tumors (Takamura et al., 2011). The observations proposed that autophagy probably has a key part in modulating formation of tumors at early stages, probably as a process that limits malignant transformation. Reports also imply that mice genetically modified to express oncogenic KRas within lung tissue, in combination with the deletion of ATG5 or ATG7 genes, demonstrate an elevated incidence of benign tumors. Significantly, these tumors do not result to malignant forms, further highlighting complex regulatory role of autophagy in advancing tumor (Guo et al., 2013; Rao et al., 2014).

Studies have identified a crucial observation that cells with Ras mutations are highly reliant on autophagic process for growth and survival. Specifically, numerous human cancer cell lines that carry activating Ras mutations display constitutive high levels of autophagy *in vitro*, suggesting metabolic adaptation (Guo et al., 2011). Autophagy dependence would be a promising therapeutic concept that arises from these studies. Autophagy and mitochondrial metabolism would then be targeted to develop novel therapeutic approaches against such aggressively behaving cancer types (Guo et al., 2011). Some reports suggest that in fibroblast cells, the activated HIF1α promotes autophagy, which further can help survive the fibroblast, and increased tumor mass and volume are also observed. Activated HIF1α also instigated expression of BNIP3L and BNIP3. Interestingly, the activated HIF1α shows a reduction of tumor mass and volume in breast cancer cells. Thus, activation of HIF1α may promote or suppress tumorigenesis based on cancer type (Chiavarina et al., 2010). A significant characteristic of metastatic cancer cells is their resistance to anoikis, a kind of apoptosis linked to inadequate adhesion to the extracellular matrix (ECM) (Taddei et al., 2012). PI3K/Akt and Ras/MAPK, growth factor pathways, are instigated in some cancer cells to evade anoikis. While autophagy-mediated cell death was first linked to anoikis, studies also corroborate the protective role of autophagy in this process (Kenific et al., 2010).

A process that is somewhat parallel to developmental process of embryo, where alteration in cellular morphologies characterized as EMT is found to impart a function in tumorigenic process (Brabletz et al., 2018). EMT is important in tumor invasion and metastasis because it allows epithelial cells to become mesenchymal cells, which increases motility as well as the ability to spread to other organs (Ribatti et al., 2020). It is also associated with the development of chemoresistance towards anticancer drugs (Zheng et al., 2015). A complicated relation exists between EMT and autophagy, as autophagy can play a dualistic role (Rojas-Sanchez et al., 2019). Reports suggest that autophagy can promote EMT in certain conditions; like in breast cancer cells, autophagy can promote EMT by instigating the TGF-β/Smad signalling pathway (Hwang et al., 2022; Hao et al., 2019). Thus, it is observed that alongside the EMT transition, elevated autophagy flux is also reported (Singla et al., 2017). Therefore, targeting autophagy inhibition can result in the blocking of EMT (Colella et al., 2019).

Given that autophagy is observed to impart a dualistic function in cancer, therapeutic targeting should be carefully designed and context-specific (Kumar et al., 2022). Enhancing autophagy at early stages of tumor development may prevent tumor initiation and progression by promoting cellular quality control and immune surveillance. In contrast, suppression of autophagy in established tumors may enhance cancer cell sensitivity to treatment and minimise the capability of cancerous cells to thrive in stressful conditions. Context and stage of cancer play an important role and must be well considered in developing autophagy-targeted therapies (Li et al., 2020).

## 7 Role of autophagy in tumor cell dormancy and immune modulation

Tumor cell dormancy can be described as wherein cells remain in quiescent state for a prolonged period of time. This dormancy is responsible for cancer recurrence and poses a hindrance in developing an effective, viable therapeutic strategy (Tufail et al., 2025). The mechanism of induction of dormancy in cancer cells is not well understood. There are many reports present that suggest the role of MAPK signalling in induction of cancer cell dormancy. The autophagy role in cellular dormancy has been shown in *C. elegans;* the developmentally quiescent larval state dauer diapause, is adopted in response to unfavourable conditions (Meléndez et al., 2003). Knockout of several autophagy genes in *C. elegans* resulted in death during dauer phase, indicating an important role of autophagy. Some reports suggest a diapause-like state in tumor cells when exposed to drugs (Rehman et al., 2021; Dhimolea et al., 2021). The study suggests diapause-like state (DLS) is autophagy-mediated. Moreover, in DLS tumor cells, MAPK pathway was observed to be activated. Mitophagy involvement is reported in cell renewal, migration, dormancy, and invasion (Smith and Macleod, 2019; Nazio et al., 2019; Jahangiri et al., 2021). A tumor suppressor, TSSC4, in glioblastoma and breast cancer inhibits formation of tumorsphere by downregulating autophagy (Chen et al., 2022).

Several signaling pathways regulate dormant and quiescent CSCs, including p53, retinoblastoma and some CDK inhibitors like p57, p27, p21 (Tiwari et al., 2024; Zeuner, 2015). Autophagy is needed to promote disseminated tumor cells (Vera-Ramirez et al., 2018; Sosa et al., 2013). In ovarian cancer, ARHI (tumor suppressor) instigates autophagy by PI3K-AKT signaling pathway inhibition, inducing tumor cell dormancy (He et al., 2015). Similarly, in colorectal cancer, downregulation of PLK4 is linked with autophagy induction and results in a dormant state *via* MAPK pathway (Tian et al., 2023). In breast cancer models, it is reported that ATG7 gene is essential for autophagy instigation in dormant tumor cells (Vera-Ramirez et al., 2018). Studies conducted on breast cancer have shown dual autophagy role in cancer dormancy. Autophagy induced by chemotherapy may facilitate tumor relapse, whereas autophagy intrinsic to cells delays tumor relapse (Aqbi et al., 2018). Autophagy inhibition can induce tumor dormancy emergence (La Belle Flynn et al., 2019). In some conditions, autophagy inhibition may prolong tumor dormancy (Aqbi et al., 2018).

Reports suggest that elevated autophagy in tumor cells can provide a pro-tumor function in tumor microenvironment (Yang et al., 2018; Katheder et al., 2017). The deficiency of autophagy in immunocompetent mouse produces an antitumor effect, but not in immune deficient mouse (Yamamoto et al., 2020; Eng et al., 2016). Autophagy is reported to increase the antigen cross-presentation by dendritic cells, a step in instigating cytotoxic T lymphocytes. Autophagosome derived from tumors sequesters ubiquitinated antigens like OVA and gp100 are engulfed by dendritic cells are then processed for MHC-I presentation priming CD8^+^ T cells (Mowers et al., 2018; Yi et al., 2012). The autophagy is crucial for this process; if autophagy is inhibited in dendritic cells, it results in downregulated MHC-I facilitated antigen presentation and thus impairing T cell response (Yang et al., 2023; De Souza et al., 2020). Autophagy promotes clearance of MHC-I through NBR1, a cargo receptor that assists tumor cells to evade immune detection. The inhibition of autophagy by chloroquine can restore the surface expression of MHC-I, resulting in increased T cell recognition (Yamamoto et al., 2020). Specific chemokines facilitate the trafficking of subsets of immune cell into the tumor microenvironment (Nagarsheth et al., 2017). The autophagy is reported to modulate the chemokines expression in tumor cell and thus can also alter the immune response by regulating migration of immune cells to tumor. In tumor associated macrophages the autophagy is induced by CCL2 and IL-6 which promotes the M2 polarization and TGF- β1 secretion resulting in metastasis (Kuo et al., 2022). For T cell survival, basal level of autophagy is vital whereas enhanced autophagy in conditions like chronic infection promotes T cell exhaustion through increased level of PD-1 (De Souza et al., 2020). Similarly, the CD8^+^ T cells, which are ATG5 deficient, shows increased glycolysis and histone methylation enhancing the production of IFN and TNF (DeVorkin et al., 2019).

## 8 Multidimensional approach to target autophagy

**FIGURE 3:**
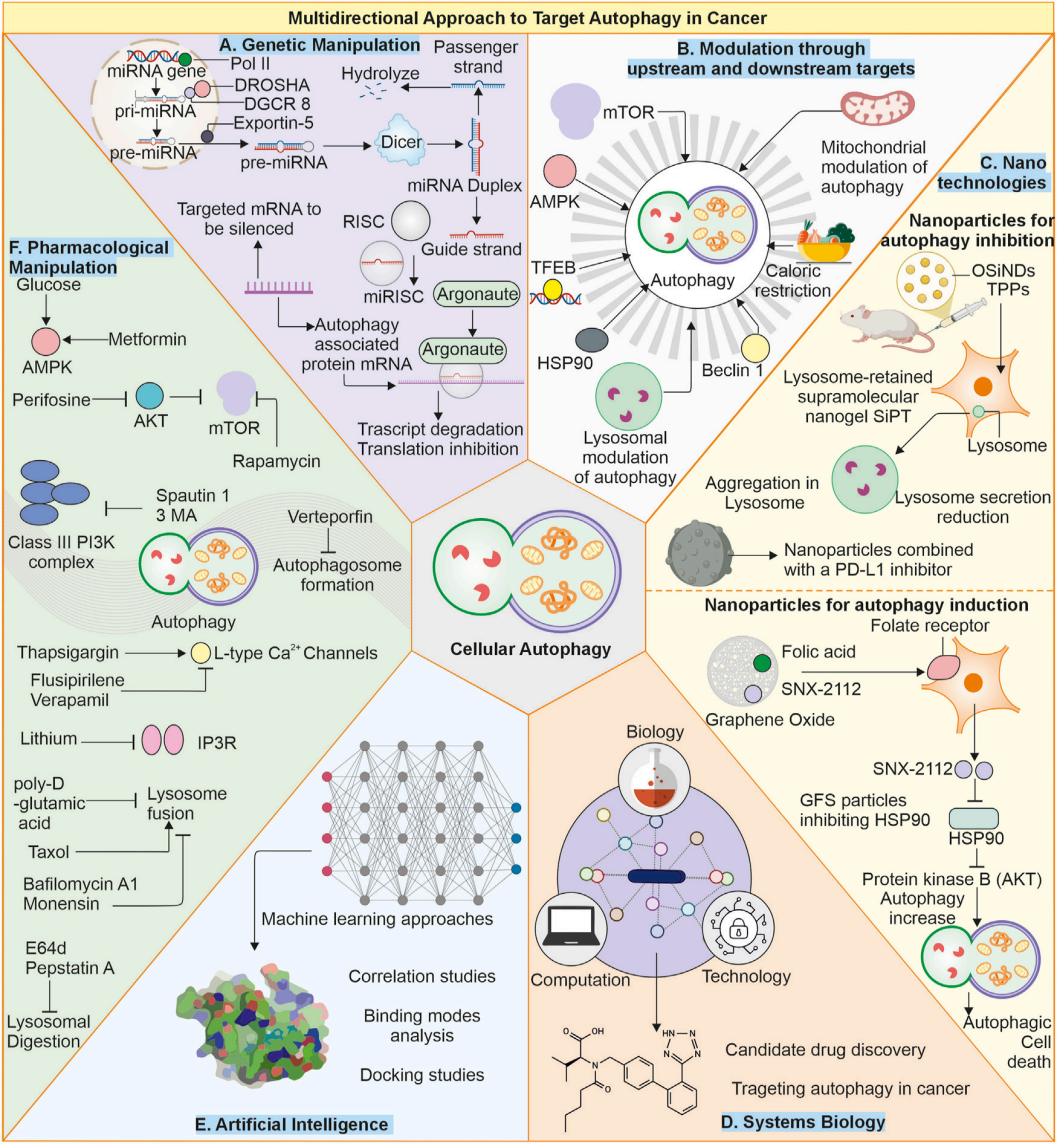
Multimodal approach to target autophagy in cancer: **(A)** Genetic manipulation: The autophagy is regulated by several autophagy-related proteins, and loss of these proteins (ATG5, ATG7) can cause suppressed autophagy, and enhanced expression of proteins like Beclin-1 can also elevate cellular autophagy. Targeting these gene targets with miRNA or gene replacement can help modulate autophagy in cells. **(B)** Modulation of autophagy through upstream or downstream targets: The Autophagy pathway can be modulated by other upstream or downstream targets of autophagy, like mTOR and AMPK. Likewise, other factors like caloric restriction chaperones and transcription factors, as well as some organelles like mitochondria, can be targeted as they can modulate autophagy levels in cells. **(C)** Nanotechnologies: Advanced nanotechnologies can help deliver specific small molecules directly to the cells, helping in modulating autophagy. **(D)** Systems biology and **(E)** Artificial intelligence: Can help identify novel targets for efficiently targeting autophagy modulation. **(F)** Small pharmacological molecules: The small molecules can directly interact with the autophagy-associated proteins or pathways and produce autophagy-modulating results. Small molecules: Metformin (Gao et al., 2020), Perifosine (Fu et al., 2009), Rapamycin (Ballou and Lin, 2008), Spautin-1 (Shao et al., 2014), 3-Methyladenine (Kosic et al., 2021), Verteporfin (Donohue et al., 2011), Thapsigargin (Nelson et al., 1994), Flusipirlene (Xia et al., 2010), Verapamil (Kania et al., 2017), Lithium (Sarkar et al., 2005), poly-D-glutamic acid (Hart et al., 1983), Bafilomycin A1 (Mauvezin and Neufeld, 2015), Taxol (Sonee et al., 1998), Monensin (Stenseth and Thyberg, 1989), E64d and pepstatin (Yang et al., 2013).

Autophagy is the primary process maintaining cellular homeostasis and stress adaptation. Under nutrient-deprivation conditions, autophagy delivers amino acids, lipids, and other crucial molecules through degrading and recycling cellular components. Autophagy’s involvement apparently provides promise in therapeutic applications to counter various complex disorders such as cancer and neuronal degenerative diseases. Both activation and inhibition of autophagy are possible. Cellular stress, starvation, and mTOR suppression stimulate autophagy (He et al., 2018), whereas several targets upstream (VPS34, Beclin 1, and ULK1 inhibitors) and downstream of autophagosome-lysosomal fusion location suppress cellular autophagy (Pavlinov et al., 2020). Some reports show that inhibition of autophagic process can cause cancer cells to be more sensitive to anticancer drugs, as it increases chemosensitivity (Yang et al., 2011). Effective targeting of modulation of autophagy can only be achieved by gaining deeper knowledge of the process involved, as represented in Figure 3.

The identification and creation of new autophagy modulators would require to explore specific molecular targets. Particular elements that are implicated in autophagic process or interact with autophagy-related proteins can serve as biomarkers for several diseases. To achieve these goals, a multimodal approach is needed where different fields of science can either combine to establish and analyse large-scale data from genomics or proteomics to develop novel therapeutic targets to modulate autophagy or develop a better delivery system for small pharmaceutical compounds for specific targeting. Another area through which autophagy may be targeted includes genetic engineering. Studies can be conducted using gene editing in the form of CRISPR-Cas9 to manipulate the genes related to autophagy to identify their functions and specific regulators (O’Prey et al., 2017). Caloric restriction as well as intermittent fasting are reported to induce autophagy and, thus, cellular wellbeing (Beaulaton and Lockshin, 1977). Such dietary regimens are proposed to work as treatments that may induce longevity and prevent age-related diseases. In summary, autophagy represents a crucial cellular mechanism indispensable for upkeeping homeostasis, adaptation to stress, and physiological functions in cells.

### 8.1 Artificial intelligence and systems biology

The artificial intelligence methodology holds the potential for accelerating drug development by predicting potential autophagy modulators and therapeutic targets-a phenomenon noted in many fields of drug discovery (Rashid, 2021). Systems biology represents a data-integrative approach that aggregates information from all tiers of biological organization-genomics, proteomics and metabolomics in an effort to build models that explain cellular processes (Wu et al., 2024). Therefore, systems biology may make it easier to outline complex networks involved in the autophagy process, making it simpler to identify regulatory nodes and potential targets for therapy. This may allow us to realize process of autophagy regulation and integration with other cellular pathways and how the alteration of autophagy might promote oncogenesis (Jegga et al., 2011; Shi et al., 2013). Machine learning, in particular, helps facilitate the analysis of such huge datasets that the studies of system biology produce. AI algorithms can predict impacts on cancer cells due to the modulated autophagy, challenging old drug paradigms and improving treatment plans. For instance, it could examine mRNA profiles of the cancers to determine which autophagy-related genes are over or under-expressed in those cancers and help tailor drugs to these specific pathways. The machine learning programs can discriminate between cancer cells by analysing immunohistochemistry images (He et al., 2020).

### 8.2 miRNA in autophagy regulation

These microRNAs (miRNA) are small non-coding RNA that modulate the levels of genes by binding to their 3′untranslated region in target mRNA, which subsequently leads to mRNA clearance or even inhibition of translation (O'Brien et al., 2018). In autophagy, miRNAs are involved, targeting key genes linked with autophagy mechanism. For instance, miR-30a targets the mRNA of Beclin 1 (BECN1), a core element of autophagy initiation complex, thus suppressing autophagy. Similarly, miR-101 targets the mRNA of RAB5A and ATG4D, which is known to induce autophagy, thereby suppressing autophagy (Zhu et al., 2009; Wang et al., 2018; Frankel et al., 2011). Such examples demonstrate the intricate network through which miRNAs regulate autophagy. In cancer, miRNAs may play either oncogenic or tumor-suppressive roles by regulating autophagy. For instance, while miR-21 is commonly overexpressed in cancers and promotes autophagy through targeting several autophagy-related genes to enhance the survival of cancer cells, miR-122 is often underexpressed in some cancers. It may regulate autophagy to inhibit tumor growth (Yan et al., 2008; Zhang et al., 2024; Saadh et al., 2024; Wang et al., 2019).

### 8.3 Autophagy modulation through nanotechnologies

Nanotechnology has now introduced novel and innovative methods of autophagy targeting, mainly with references to cancer treatment. The nanoparticle designs lead toward the targeted delivery of autophagy-modulating drugs, thus providing increased efficacy and reduced side effects for the treatments. Targeted delivery ensures that a therapeutic agent reaches a point of action while enhancing its impact at the same time and maintaining minimum levels of injury to tissues (Paskeh et al., 2022). Several studies have reported phototherapy and autophagy relations. Phototherapy can cause the instigation of autophagy and suppress cancerous cell apoptosis. Studies have employed autophagy inhibitors to sensitize tumor cells to phototherapy. One method reported is to disrupt lysosomes with a nanogel (SiPT) containing organosilica nanodots (OSiNDs), photosensitizer TPPS, and PEG-PLE. SiPT accumulates in lysosomes, and TPPS produces ROS under 532 nm laser irradiation, promoting tumor cell apoptosis and inhibiting autophagy through lysosome destruction. This combined approach blocks autophagy, hence making phototherapy more effective. Despite the enhanced localization of tumors, SiPT still retains distribution in other parts, which explains the need for targeting strategies (Zhang X. et al., 2020). Similarly, nanoparticles loaded with PD-L1 inhibitors are also a therapeutic strategy (Tran and Phuong Tran, 2022). A study reported that the formulation of folic acid, graphene oxide (GO), SNX-2112, is a newly designed GFS. The effect of this is that when under 808 nm laser light (NIR), GFS releases SNX-2112 to inhibit HSP90, thereby causing death from autophagy. Flow cytometry reports indicated that a combination of NIR and 3-MA-based autophagy inhibition further enhanced cell death (Deng et al., 2020).

## 9 Pharmacological and other interventions in targeting autophagy in cancer

Pharmacological agents targeting to modulate autophagy are one of the potential options in cancer treatments. Small molecules influence autophagy by targeting autophagy proteins directly or indirectly. Autophagy has a dual nature, which was realized during several studies of cancer biology, and its role depends on either stage or type of cancer (Yun et al., 2020). One of the approaches used when targeting autophagy in chemotherapy is the use of inhibitory drugs for autophagy. Hydroxychloroquine and chloroquine are well-known inhibitors of autophagy. These inhibitors prevent fusion of the autophagosome and lysosome, effectively preventing clearance of contents of autophagic vesicle. Inhibition of autophagy sensitises cancerous cells to chemotherapy and radiation stress and damage (Mauthe et al., 2018a). The rationale behind the therapy is that blocking the protective process of autophagy reduces the cancer cell’s capability to respond to the stressful environment resulting from the treatment, and it will increase cell death, thereby improving the outcomes. It is also to be noted that when insulin and amino acids are present, autophagy process is inhibited (Liu et al., 2009). Another important class of drugs in this regard is mTOR inhibitors. The mTOR is a crucial negative autophagy modulator. Drugs such as rapamycin and its derivatives, known as rapalogs, inhibit mTOR, thereby activating autophagy (Ballou and Lin, 2008). Molecules like everolimus, Torin 1 and Temsirolimus are also mTOR inhibitors (Witzig et al., 2015; Thoreen et al., 2009; Malizzia and Hsu, 2008). In some cases, excessive autophagy stimulation can result in autophagy-dependent cell death, which could be beneficial in some cases. This would create a metabolic stress environment that is detrimental to the survival of the cancerous cells (Fulda and Kögel, 2015).

Moreover, in some conditions, autophagy activators act as therapeutic agents. Activation of AMPK by small molecules like metformin drugs can induce autophagy (Goel et al., 2022). AMPK functions as an energy sensor to degrade cellular components and make the cells generate more energy at a low cellular level (Hardie et al., 2012). Therefore, autophagy activators can contribute towards the inhibition of growth by enhancing clearing and recycling of damaged cellular constituents. This strategy may particularly be significant in cancers in the initial stages as enhanced autophagy can help limit inflammation and genetic instability, induce cell death in transformed cells and uphold homeostasis (Yang et al., 2011).

**FIGURE 4:**
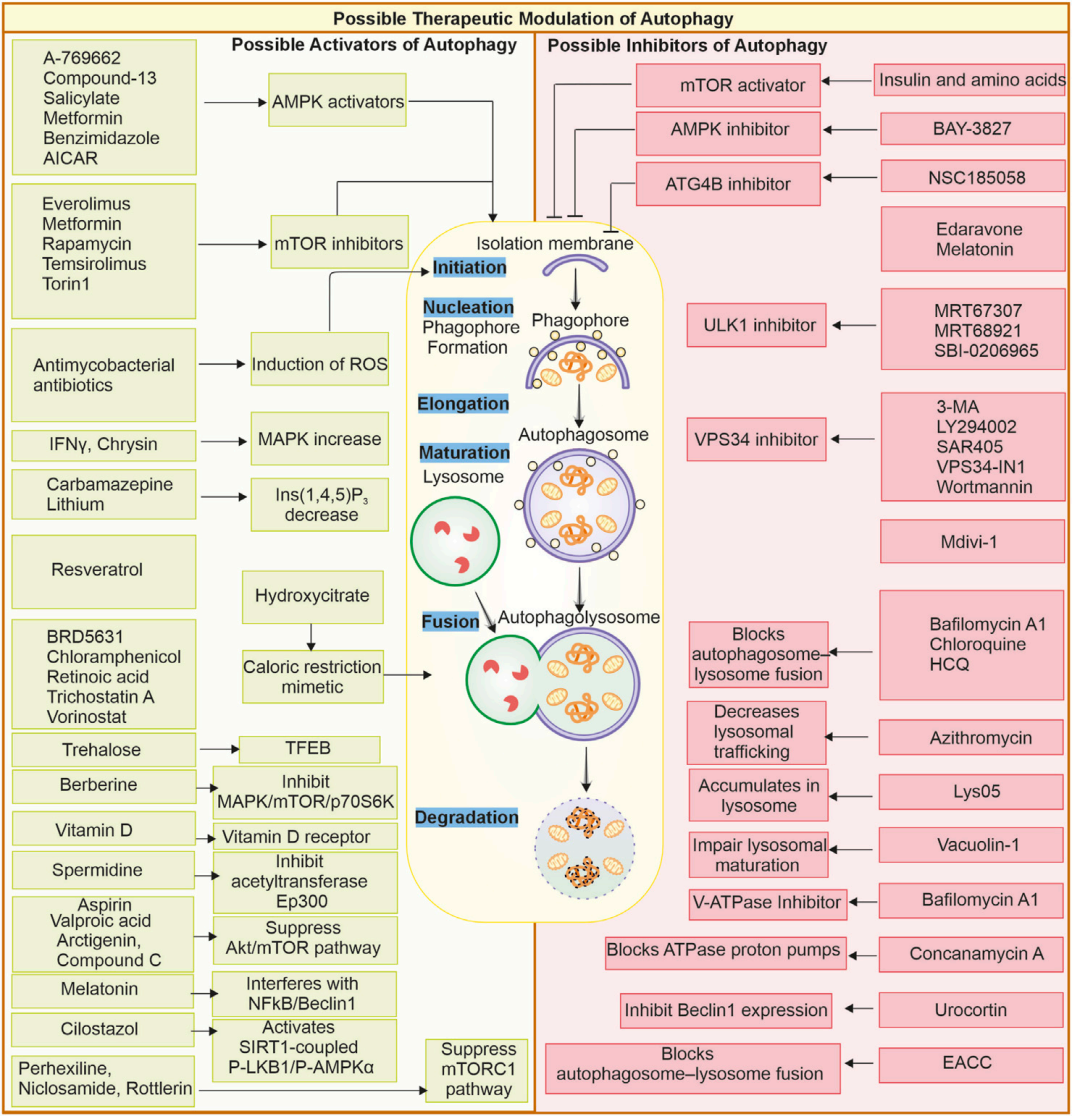
Possible therapeutic modulation of autophagy through small molecules: Small molecules can modulate autophagy; they can either be inhibitors or promoters. Some of the activators and inhibitors are represented. Autophagy activators: A-769662 (Walter et al., 2016), AICAR, benzimidazole, Salicylate, Compound-13 (Kim et al., 2016), Everolimus (Haas et al., 2019), Metformin, Rapamycin (Kim and Guan, 2015), Temsirolimus, Torin1 (Chandrika et al., 2017), Melatonin (Das et al., 2024), Antimycobacterial antibiotics (Kim et al., 2012), Chrysin (Jung et al., 2022), IFN-γ (Matsuzawa et al., 2012), Carbamazepine (Adikesavalu et al., 2021), Lithium (Sarkar et al., 2005), Hydroxycitrate (Piffoux et al., 2021), Resveratrol, Spermidine (Morselli et al., 2011), BRD5631 (Kuo et al., 2015), Chloramphenicol (Hsu et al., 2019), Retinoic acid (Rajawat et al., 2010), Trehalose (Rusmini et al., 2019), Trichostatin A (Liu et al., 2021), Vorinostat (Patra et al., 2022) Arctigenin (Jiang Y.a. et al., 2020), Aspirin (Peng et al., 2023), Compound C (Vucicevic et al., 2011), Berberine (Zhang Q. et al., 2020), Vitamin D (Wu and Sun, 2011), Valproic acid (Xia et al., 2016), Cilostazol (Park et al., 2016), Perhexiline, Niclosamide, Rottlerin (Balgi et al., 2009). Autophagy inhibitors: Amino acids and insulin (Shen et al., 2021), BAY-3827 (Hawley et al., 2023), NSC185058 (Ryan et al., 2024), Edaravone (Yin et al., 2019), Melatonin (Luo et al., 2020; Boga et al., 2019), MRT67307, MRT68921 (Petherick et al., 2015), SBI-0206965 (Knudsen et al., 2020; Egan et al., 2015), 3-MA (Heckmann et al., 2013; Wu et al., 2010) LY294002 (Wang et al., 2016; Blommaart et al., 1997), SAR405 (Pasquier, 2015), VPS34-IN1 (Meunier et al., 2020), Wortmannin (Yang et al., 2013; Takatsuka et al., 2004), Mdivi-1 (Aishwarya et al., 2020), Chloroquine, HCQ (Maycotte et al., 2012; Mukhopadhyay et al., 2011; Mauthe et al., 2018b), Lys05 (McAfee et al., 2012), Azithromycin (Takano et al., 2023), Concanamycin A, Bafilomycin A1 (Mauvezin and Neufeld, 2015) (Yano et al., 2015), Vacuolin-1 (Lu et al., 2014), Urocortin (Valentim et al., 2006), EACC (Vats and Manjithaya, 2019).

Combination therapies have become a useful tool for making targeted therapy against cancer effective. The addition of autophagic process inhibitors process combined with other anticancer treatments overcomes the mechanisms of drug resistance and thus leads to improving patient outcomes (Mele et al., 2020). Thus, chloroquine combined with chemotherapy or targeted therapy is highly effective in making the protective autophagy pathway of cancer cells cytotoxic effects. In other words, this synergistic approach utilises the strengths of more than one therapy to kill cancerous cells more efficiently whilst limiting the chances of treatment resistance (Varisli et al., 2020; Agalakova, 2024). Another therapeutic approach is to use novel small-molecule compounds that target proteins associated with autophagy. These compounds could modulate autophagy with much greater precision and much fewer side effects than broad-range drugs. For instance, SBI-0206965, a ULK1 inhibitor, affects autophagy by targeting specific components of the machinery of autophagy; it is also reported to inhibit AMPK, which offers a potential for more effective and targeted use of cancer therapies (Knudsen et al., 2020). Similarly, other compounds like MRT67307 and MRT68921 are also ULK1 and ULK2 inhibitors (Petherick et al., 2015). Several other small molecules and drugs can target various autophagy-related proteins; they may induce or suppress autophagy flux by acting at different stages and steps of autophagy, as shown in Figure 4.

Some specific autophagy inhibitors are chloroquine and hydroxychloroquine, these compounds work by blocking fusion between autophagosome and lysosome through alkalinizing the lysosomes, which further reduces autophagy flux. The advantage of such compounds is that they are FDA-approved for use in cases of malaria and autoimmune disease; thus, repurposing such drugs can accelerate the clinical approval process (Mao et al., 2018; Verbaanderd et al., 2017). Another advantage of such drugs is that they have broad anticancer activity. CQ and HCQ are reported to be effective in various cancers like pancreatic, glioblastoma, esophageal, and breast, causing cell death (Cai et al., 2018). These drugs are also reported to be utilised in combination therapy and increase the efficiency of chemotherapy by overcoming the hypoxia induced resistance (Verbaanderd et al., 2017). There is also some evidence suggesting these drugs can restore MHC-1 expression in pancreatic cancer (Yamamoto et al. 2020; Mauthe et al., 2018a). There are few limitations of these drugs as they may have non desired effects like disruption of Golgi and at higher doses, can cause retinal damage (Yusuf et al., 2023; Mauthe et al., 2018a). Lys05 is a dimeric derivative of chloroquine that can accumulate in the lysosome and inhibit autophagy. This compound has some advantages, like higher potency than CQ in inhibiting autophagy and decreasing tumor growth. Under hypoxic conditions can target acute myeloid leukemia by suppressing mitophagy and impairing mitochondria. It can be used in a combination therapy with cytarabine and azacytidine in AML, clearing CD34^+^CD38 leukemic stem cells. There are some limitations of this compound; its high dose causes intestinal toxicity (Amaravadi and Winkler, 2012; Wang et al., 2024). Specific autophagy inhibitors target autophagy molecules; ULK-101, an autophagy inhibitor that inhibits specifically the ULK1 kinase and interrupts the autophagy initiation. Some advantages of such inhibitors are that they suppress the formation of autophagosomes, avoiding any compensatory mechanism observed with lysosomal inhibitors. ULK-101 shows reduced off-target effects and can be used in combination with nutrient deprivation in some KRAS mutant lung cancer. There is limited clinical data present when compared to CQ (Martin et al., 2018).

These developments emphasise the requirement for disentangling autophagy molecular mechanisms of toward novel and accurate therapeutic modalities. Gene therapy has emerged as another promising avenue for directly modulating the autophagy pathway in cancer by introducing genetic material that is known to either enhance or impair autophagy. There are several autophagy genes reported to be either mutated in diseases or implicated in complex diseases (Levine and Kroemer, 2019; Koveitypour et al., 2019; Amaravadi et al., 2019). This strategy may be applied to treat cancers with particular genetic alterations in autophagy-related genes. For example, restored mutated or downregulated autophagy-related genes in cancer cells can serve to enhance autophagic cell death and inhibit cancer development. This approach may enhance the efficacy of gene manipulation for targeted and highly efficient cancer therapy.

Nano technology’s drug delivery system has innovative targeting schemes to enhance the drug’s ability to target autophagy pathways to cancer therapy. Nanoparticles can be engineered to target the cells directly by delivering autophagy modulators. This means that the treatments will have a higher efficacy and lower side effects. For example, nanoparticles might be engineered to deliver autophagy inhibitors, which would then be controlled at the tumor site. Such a targeted delivery system would enable therapeutic agents to be delivered to the intended site of therapeutic action, hence increasing their effectiveness while reducing the adverse impact on other normal tissues. This is an approach that combines nanotechnology with the therapeutic value of modulating autophagy to offer advanced options for the treatment of cancer (Paskeh et al., 2022). Some dietary supplements and natural compounds have also been proven to modulate autophagy. For instance, resveratrol is a polyphenol contained in grapes and red wine; it activates autophagy through the AMPK pathway (Pasinetti et al., 2015). Spermidine, the naturally occurring product in diverse food sources, seems to favour the autophagy process with links to increased lifespan from model organism studies (Satarker et al., 2024; Hofer et al., 2024). Consumption of these compounds as supplements could improve levels of activity for autophagic machinery to promote cell health. Adding these natural compounds to cancer therapy will enable the investigation of new and complementary ways of modulating autophagy, thus leading to better treatment outcomes. Other precise ways of targeting autophagy-related genes are through the use of antisense oligonucleotides (ASOs). ASOs are small, nucleic acids strands (synthetic) that will bind to the target gene’s mRNA and halt protein synthesis, thus modulating autophagy activity (Dhuri et al., 2020; Hung et al., 2024). This approach allows a tight control over expression of autophagy-associated genes and hence may be used for promoting or inhibiting the processes of autophagy within the cancer cells. By focusing on specific genes in the autophagy pathway, ASOs offer the power to modulate and thus develop targeted cancer therapies against such diseases.

## 10 Conclusion and future outlook

Autophagy is a cellular degradative process that clears out worn-out proteins and organelles. It maintains cellular homeostasis as well as genomic stability by clearing out cellular toxic waste and regulating ROS levels. Autophagy is also implicated in coordinating with the immune system. Autophagy is also implicated in metabolism modulation. It is a critical mechanism in cells activated during stress response and is mainly a pro-survival process. Autophagy is also required to replenish the amino acid pools during starvation by recycling the cellular organelles and proteins. Impaired autophagy in several diseases, like neurodegenerative diseases and in different cancers, has been reported. In diseases like cancer, autophagy can perform a complex dualistic function. In initial stage of tumor formation, autophagy acts as a cellular protector, clears out cell debris, maintains genomic stability, and may clear out initial transforming cells, thus acting as a tumor suppressor. Its evidence is present when autophagy-associated proteins like ATG5, ATG7, and Beclin-1 are impaired, and then spontaneous tumors are reported, suggesting their tumor suppressive function. Meanwhile, in many cancerous cells, heightened levels of autophagy and autophagy-related proteins are reported to help cancer cells survive stresses like hypoxia and oxidative stress. Autophagy also provides cancer cells chemoresistance against anticancer drugs, thus functioning as tumor-supportive.

This makes autophagy a lucrative target for developing therapeutics against cancer. Autophagy is a multistep process that requires several molecules for regulation; thus, all these regulatory molecules and steps can be used to modulate autophagy. There are several reported pharmacological drugs that inhibit autophagy and present multiple approaches to cancer therapy. Autophagy can be modulated through autophagic inducers or inhibitors and novel small-molecule anti-cancer agents designed to instigate apoptosis in cancerous cells as well as increase the therapeutic efficacy of the main treatment strategies. Genetic therapies, nanoparticle delivery systems, dietary supplements, antisense oligonucleotides, and high-throughput screening methods have also been utilized to suppress autophagy in cancer. Personalized medicine approaches allow for the refinement of targeted therapies directed at autophagy regulation, tailored to the patient-specific characteristics. The personalized approaches, in conjunction with continuous clinical research, have the potential to improve treatment approaches and further enhance the patients’ outcomes. The progress in our understanding of autophagy biology will be the foundation of innovative and effective therapies in the field of oncology. Various pharmacological interventions are likely to play a very important role.

High-throughput cell-based assays are essential tools for screening of large libraries of compounds to identify potential autophagy modulators. It can be used to evaluate the impact of various compounds on the autophagic process by real-time measurements of autophagy flux. The rise in drug development and the introduction of new cancer treatments are increased by identifying new drugs that either promote or inhibit the autophagy process. The strategy will thus utilise advanced screening technologies coupled with deep autophagy biology insights to select some promising candidates. In this approach, personalised medicine allows the stratification of patients according to genetic and molecular profiling, selecting only those with the potential for benefit. For instance, tumors with a high basal autophagy rate or carrying particular genetic alterations in autophagy-related genes are probably more susceptible to inhibition of autophagy. Personalised medicine through tailored treatment could thus provide a better therapeutic response and reduce the risk of drug resistance. Thus, multidimensional approaches can be developed to advance research in this field in the future.

## Glossary

3-MA: 3-methyladenine
AICAR: 5-Aminoimidazole-4-carboxamide ribonucleotide
AMPK: AMP-activated protein kinase
ATG: autophagy related
BAG: Bcl2-associated athanogene
Bcl2: B-cell leukemia/lymphoma 2
BIF1: Bax-interacting factor 1
CDK: Cyclin-dependent protein kinase
CMA: Chaperone-mediated autophagy
CQ: chloroquine
CSCs: Cancer stem cells
eMI: endosomal microautophagy
EMT: epithelial to mesenchymal transition
ER: endoplasmic reticulum
ESCRT: endosomal sorting complex required for transport
FIP200: Focal adhesion kinase family interacting protein of 200 kD
HCQ: hydroxychloroquine
HIF1: Hypoxia inducible factor 1
Hip: Hsc70-interacting protein
Hop: Hsc70-Hsp90 organizing protein
HSP: heat shock protein
ILV: intralumenal vesicles
KFERQ: Lysine, phenylalanine, Glutamic acid, Arginine, Glutamine
LAMP-2A: Lysosome-associated membrane protein type 2a
LC3: Microtubule-associated protein light chain 3
LE/MVB: late endosomes/multivesicular bodies
mTOR: mechanistic target of rapamycin
PE: phosphatidylethanolamine
PEG-PLE: methoxy-poly (ethylene glycol)-poly (L-glutamic acid sodium salt) (PEG-PLE)
PI3K: Phosphoinositide 3-kinase
PI3P: Phosphoinositide-3-phosphate
SENDA: static encephalopathy of childhood with neurodegeneration in adulthood
SQSTM1: sequestosome
TCA: tricarboxylic acid cycle
TME: tumor microenvironment
TPPS: tetraphenylporphinesulfonate
ULK1: unc-51-like autophagy activating kinase 1
UVRAG: Ultraviolet radiation resistance-associated gene.
